# Supplementary material for: A comparison of self-triage tools to nurse driven triage in the emergency department
Source: PLoS One. 2024 Aug 28;19(8):e0297321. doi: 10.1371/journal.pone.0297321 (PMC11356451; doi:10.1371/journal.pone.0297321)
Supplement: Attachment — Submitted filename: Response to Reviewers July.docx [file pone.0297321.s002.docx]

July 8, 2024

Dear Dr. Sebastian Schnaubelt and the PLOS ONE Reviewer team,

In follow-up to the revisions sent previously, the dataset has now been uploaded to the Harvard Dataset repository. It is also attached as a supplementary file as I had done previously. The link to the Harvard Dataset repository is here: <https://dataverse.harvard.edu/dataset.xhtml?persistentId=doi:10.7910/DVN/MGDOWQ>.

Thank you for your continued interest in our study.

Sincerely,

Sachin V Trivedi, Rachit Batta, Nicolas Henao – Romero, Prosanta Mondal, Tracy Wilson and James Stempien

Reviewer's Responses to Questions

**Comments to the Author**

1. Is the manuscript technically sound, and do the data support the conclusions?

The manuscript must describe a technically sound piece of scientific research with data that supports the conclusions. Experiments must have been conducted rigorously, with appropriate controls, replication, and sample sizes. The conclusions must be drawn appropriately based on the data presented.

Reviewer #1: Yes

Reviewer #2: Yes

Thank you for your feedback

2. Has the statistical analysis been performed appropriately and rigorously?

Reviewer #1: Yes

Reviewer #2: No

See below with response to individual comments

3. Have the authors made all data underlying the findings in their manuscript fully available?

The [PLOS Data policy](https://can01.safelinks.protection.outlook.com/?url=http%3A%2F%2Fwww.plosone.org%2Fstatic%2Fpolicies.action%23sharing&data=05%7C02%7Csachin.trivedi%40usask.ca%7C1133eb51ec854cc7089708dc78118316%7C24ab6cd0487e47229bc3da9c4232776c%7C0%7C0%7C638517263805934474%7CUnknown%7CTWFpbGZsb3d8eyJWIjoiMC4wLjAwMDAiLCJQIjoiV2luMzIiLCJBTiI6Ik1haWwiLCJXVCI6Mn0%3D%7C0%7C%7C%7C&sdata=x2QhpRmuNYOiMEBmTQr%2FYPtlUeGsaP1k%2BajtHZWFOCc%3D&reserved=0) requires authors to make all data underlying the findings described in their manuscript fully available without restriction, with rare exception (please refer to the Data Availability Statement in the manuscript PDF file). The data should be provided as part of the manuscript or its supporting information, or deposited to a public repository. For example, in addition to summary statistics, the data points behind means, medians and variance measures should be available. If there are restrictions on publicly sharing data—e.g. participant privacy or use of data from a third party—those must be specified.

Reviewer #1: Yes

Reviewer #2: No

See paragraph in above letter

4. Is the manuscript presented in an intelligible fashion and written in standard English?

PLOS ONE does not copyedit accepted manuscripts, so the language in submitted articles must be clear, correct, and unambiguous. Any typographical or grammatical errors should be corrected at revision, so please note any specific errors here.

Reviewer #1: Yes

Reviewer #2: Yes

Thank you for this feedback

5. Review Comments to the Author

Please use the space provided to explain your answers to the questions above. You may also include additional comments for the author, including concerns about dual publication, research ethics, or publication ethics. (Please upload your review as an attachment if it exceeds 20,000 characters)

Reviewer #1: Thank you for asking me to review the manuscript PONE-D-23-43949, A comparison of self-triage tools to nurse driven triage in the emergency department

The statistical analyses are appropriate. The style of the paper is appropriate according to the journal’s requirements. Appreciate the authors best efforts for testing a self-triage tool due to increasing digitalisation and the use of artificial intelligence; no further comments

Thank you for this feedback.

Reviewer #2: Thanks for the opportunity to review this novel study

Abstract
• The abstract provides a concise overview of the study's purpose, methods, and key findings. However, it could be enhanced by briefly stating the significance of the findings in the context of existing literature or potential implications for emergency department operations.
• Include a sentence on the implications of the findings and a brief comparison with existing literature to contextualize the significance of the study.

The conclusion section in the abstract has been updated to comment that self-triage tools applied to a general ED population may not be viable, but it does make mention that there is some success with self-triage tools which have been designed for specific subset of presenting complaints.

Background
• The introduction clearly establishes the background, but I am not clear on the “need” for this study. What is the cost or downside of the current model? References are well integrated to justify the research.

A comment surrounding the current model of triage, and how it’s efficiency can be improved by having the patient provide subjective information prior to the triage nurses objective assessment has been added.

• The introduction could benefit from a more detailed discussion on the limitations of previous studies and how this study aims to address them.

The introduction has been updated to address this. In particular, we comment on how this study builds upon our previous work.

Methods
• The description of the methodology is detailed, including sample selection, intervention tools, and data collection processes which are crucial for replicability.

Thank you for this comment

• There is a clear explanation of the statistical methods used, which is appropriate for the study design. However, would an agreement study be a better design? Who is to say what the right triage category is? It would be interesting to link the scores (AGST and CTAS) to an objective marker of ED clinical needs.

As a pilot study testing the viability of this new innovation, we chose to look at agreement by way of the Kappa statistic (to assess inter-rater reliability between these self-triage tools and the goal standard of nurse-driven triage) as well as the descriptive rates of agreement, over-triage, or under-triage as described in our manuscript. We felt that this would be the best method as a “proof-of-concept,” which, if successful, would have lent itself to a more robust follow-up project where we expanded the presenting complaint options.

The question of “who is to say what the right triage category is?” is a valid one. Although the triage process is aided by an objective physical exam assessment by a triage nurse, it is inherently challenged by variability as we refer to in references 20 and 21 in the manuscript. Triage is certainly an imperfect process which serves to more or less establish the order in which patients are seen. The purpose of this study was to test these innovations to see how they can evolve the existing triage process. Though we agree that looking at how any triage score (both self-triage generated or otherwise) is correlated with ED clinical needs and resource utilization, this was outside the scope of our study. Looking at how triage scores correlate to ED specific key-performance indicators and utilization is an interesting idea for another way to improve triage.

Results
• The results are presented in a clear, logical order. Tables are appropriately used to summarize data, making it easy to understand.

Thank you for this comment

• The study does well to present the quantitative data; however, there is a lack of depth in discussing unexpected results or discrepancies.
• What are the reasons behind the poor agreement levels of the self-triage tools compared to CTAS? Could you run some analysis to shine light on this?

We have tried to leave only the objective results in the results section. We purposefully left commenting on the unexpected results to the discussion, where we speak to our subjective interpretation of results and references to existing literature. As mentioned in our discussion, our results are seemingly in line with existing literature (previous studies have shown high rates of over-triage, and we had this as well as under-triage in our results). We have also commented in the limitations section how we believe that over-triage may have come from patients hoping to expedite their assessment within the ED.

Discussion
• The discussion contextualizes the results within the broader literature and discusses potential reasons for the findings. It also acknowledges the study's limitations and suggests areas for future research.

Thank you for this comment

• This is a convenience sample – a limitation that should be explored in more detail.

This section has been expanded on within the discussion of limitations

• More than 6 % left before they had a CTAS – this seems very high. Did the intervention cause this level of left before seen or is this the base rate at your facility?

This is in line with our existing rate of patients who leave without being seen. Unpublished rates of patients leaving without being seen from 2022-2023 are as high as 8.8%, and currently for 2023-2024 these are as high as 13.6%. We do not believe that our intervention introduced additional factors prompting patients leaving without being seen. We have now made a note of the patients leaving without being seen in the manuscript.

• The discussion remains consistent with the results, and does not overstate the findings.

Thank you for this comment

• Suggest providing more detailed suggestions for future research directions and potential practical applications of the study findings in real-world settings.

This has been expanded on with a line about artificial intelligence, and we will also separate this section under a separare conclusion heading as per the below comment

Conclusion
• Please separate out from the discussions

As above, this is now done
